# A Grey Wolf Optimizer Algorithm for Multi-Objective Cumulative Capacitated Vehicle Routing Problem Considering Operation Time

**DOI:** 10.3390/biomimetics9060331

**Published:** 2024-05-30

**Authors:** Gewen Huang, Yuanhang Qi, Yanguang Cai, Yuhui Luo, Helie Huang

**Affiliations:** 1 Information and Network Center, Jiaying University, Meizhou 514015, China; huang_gewen@163.com; 2 School of Automation, Guangdong University of Technology, Guangzhou 510006, China; caiyg99@163.com (Y.C.); luoyh2020@163.com (Y.L.); 3 School of Computer Science, University of Electronic Science and Technology of China, Zhongshan Institute, Zhongshan 528402, China; 4 Guangdong Science and Technology Infrastructure Centre, Guangzhou 510033, China; huanghl@gdcc.com.cn

**Keywords:** dynamic grey wolf optimizer algorithm, multi-objective, cumulative capacitated vehicle routing problem, emergency logistics, floating 2-opt

## Abstract

In humanitarian aid scenarios, the model of cumulative capacitated vehicle routing problem can be used in vehicle scheduling, aiming at delivering materials to recipients as quickly as possible, thus minimizing their wait time. Traditional approaches focus on this metric, but practical implementations must also consider factors such as driver labor intensity and the capacity for on-site decision-making. To evaluate driver workload, the operation times of relief vehicles are typically used, and multi-objective modeling is employed to facilitate on-site decision-making. This paper introduces a multi-objective cumulative capacitated vehicle routing problem considering operation time (MO-CCVRP-OT). Our model is bi-objective, aiming to minimize both the cumulative wait time of disaster-affected areas and the extra expenditures incurred by the excess operation time of rescue vehicles. Based on the traditional grey wolf optimizer algorithm, this paper proposes a dynamic grey wolf optimizer algorithm with floating 2-opt (DGWO-F2OPT), which combines real number encoding with an equal-division random key and ROV rules for decoding; in addition, a dynamic non-dominated solution set update strategy is introduced. To solve MO-CCVRP-OT efficiently and increase the algorithm’s convergence speed, a multi-objective improved floating 2-opt (F2OPT) local search strategy is proposed. The utopia optimum solution of DGWO-F2OPT has an average value of two fitness values that is 6.22% lower than that of DGWO-2OPT. DGWO-F2OPT’s average fitness value in the algorithm comparison trials is 16.49% less than that of NS-2OPT. In the model comparison studies, MO-CCVRP-OT is 18.72% closer to the utopian point in Euclidean distance than CVRP-OT.

## 1. Introduction

In past decades, natural disasters like Cyclone Nargis in Myanmar (2008) and the Sichuan earthquake in China (2008) have wreaked havoc on communities worldwide, causing significant loss of life and economic damage [1]. A United Nations report [2] estimated that between 1998 and 2017, climate-related disasters claimed the lives of 1.3 million people and affected 4.4 billion through injuries, displacement, or the urgent need for assistance. More than 500 earthquakes, including associated tsunamis, accounted for 56 percent of the total deaths, with an economic toll reaching a staggering USD 290.8 billion. The United Nations Office for Disaster Risk Reduction estimates that the total cost caused by disasters, including earthquakes and tsunamis, is between USD 250 billion and USD 300 billion a year. Such figures bring the imperative nature of effective emergency operation management to the forefront, especially the crucial role of minimizing losses and casualties and ensuring the survival and recovery of those affected [3]. Immense destruction, the vast affliction of casualties, and massive economic losses often accompany these sudden disasters. Therefore, concerted efforts toward establishing timely, efficient rescue measures and executing proficient emergency relief distributions are paramount. In the aftermath of disasters, optimizing vehicle routing is a key factor in ensuring the efficient distribution of emergency supplies, thus enhancing disaster response operations and aiding in restoring normalcy for affected communities. Meanwhile, factors such as the promptness and equitability of material distribution processes become critical, with a particular focus on the timing of relief materials reaching each disaster site [4]. Traditional objectives centered on cost minimization do not adequately capture the transportation demands of these critical scenarios. As a response, the concept of “cumulative wait time” [5,6,7] has emerged in the literature, essentially conveying the average wait time experienced by all disaster-affected locations. This measure serves as an index of fairness, ensuring that the relief operations systematically address the urgency of needs across all impacted sites [8].

The cumulative capacitated vehicle routing problem (CCVRP) addresses the optimization of customer wait times and was first introduced by Ngueveu et al. [5] in 2010. This problem formulation aligns with the requirements for prompt logistics distribution, finding relevance in diverse sectors like disaster relief and medical supplies. Subsequent research has expanded on this variant of VRP. Nucamendi-Guillén et al. [9] studied CCVRP and used an iterative greedy algorithm to solve large-scale instances. Rivera et al. [10] proposed the multi-trip CCVRP and solved a small-scale instance using mixed-integer linear programming. To improve the rapidity and fairness of emergency rescue, Zeng et al. [11] established the model of multi-depot CCVRP. For a situation in which a vehicle can perform multiple journeys to serve a group of disaster-affected points in emergency logistics, Rivera et al. [12] studied the multi-trip cumulative capacitated single-vehicle routing problem and proposed two mixed-integer linear programming models, a flow-based model and a set partition model for small-scale instances of 20 stations. Pei et al. [13] proposed and solved the cumulative heterogeneous fleet emergency vehicle routing problem with demand weighting. Lalla-Ruiz et al. [14] proposed and solved the multi-depot cumulative capacitated vehicle routing problem for a situation in which distribution vehicles may start from different places. Ke [15] proposed a brain storm optimization algorithm for solving CCVRP and designed aggregation and divergence operations. Sze et al. [16] used large neighborhood search as a decentralized strategy and proposed a two-stage adaptive variable neighborhood search algorithm for solving CCVRP. Kyriakakis et al. [17] implemented an ant colony system-variable neighborhood decent and max-min ant system-variable neighborhood decent for the solution of the cumulative capacitated vehicle routing problem. Literals [18,19,20] are devoted to the solution of CCVRP with time windows, which consider the constraints that customers must serve within the time window and aim at minimizing the wait time of all customers. Farzadnia et al. [21] introduced the cumulative school bus routing problem, the objective of which is to select a drop-off point for each student among potential locations within a certain walking distance and generate routes such that the sum of the arrival times of all students from their school to their homes is minimized.

The risks associated with the state of the transport network after a disaster cannot be ignored, for example, disasters may make the transport network unreliable, and secondary disasters may make the road network worse. These conditions also increase the work stress of transport vehicle drivers. Therefore, in order to perform these rescues for the health of the personnel and reduce the risk of transport, it is necessary to minimize the overtime operating time of the vehicles. As a whole, it is necessary to balance the workload between different vehicles in the fleet [22]. Li et al. [23] addressed the vehicle routing problem with workload balance (VRPWB) and micro-cluster-based VRPWB to minimize the total traveling costs and balance the workload. In [24], the vehicle routing problem with time windows and route balance (VRPTWRB) was established, in which reasonable workload resources and equity functions are selected. In [25], multi-objective CVRP with workload balancing was studied, achieving the optimized result in which the equitable workload is assigned for each vehicle.

Recent developments in logistics, especially in disaster relief operations, underscore the necessity of incorporating multiple objectives into decision-making processes. This shift acknowledges the complex realities on the ground, urging the design of models and algorithms that output diverse optimization strategies for logistics transportation. Reflecting this paradigm shift, contemporary scholarly efforts have pivoted toward multi-objective vehicle routing problems. Several scholars [26,27,28,29] developed multi-objective programming models for the Green Vehicle Routing Problem to handle the economic and environmental objectives simultaneously. To deal with the complexity of charging requirements, some multi-objective VRP models for electric vehicle were exploited [30,31]. Liang et al. [32] modeled the perishable delivery problem as a bi-objective vehicle routing problem with multiple time periods, aiming to minimize transportation costs and maximize customer satisfaction by minimizing the loss of perishable freshness. Menares et al. [33] proposed a mixed-integer linear programming model for the bi-objective time-dependent vehicle routing problem with delivery failure probabilities, in which operational costs and delivery failure rates were jointly minimized. Soriano [34] modeled the multi-depot vehicle routing problem with profit fairness as a bi-objective optimization problem, which included a fairness objective function and a cost minimization function. In [35], the bi-objective perishable product delivery routing problem with stochastic demand was solved to minimize the total expected operating cost and maximize the total expected customer satisfaction. In [36], a tri-objective mathematical model was proposed for the collaborative multicenter vehicle routing problem, with time windows and defaulting member withdrawal. In [37], a multi-depot vehicle routing problem with time windows and three-dimensional loading constraints is formulated as a bi-objective mixed-integer programming model to minimize the total operating costs while maximizing the average loading rate of vehicles. Yin [38] addressed multi-objective optimization for the vehicle routing optimization problem in low-carbon intelligent transportation, which aimed to reduce the energy consumption and carbon emissions generated during urban logistics transportation and distribution. In [39], a new multi-objective MILP model combining a two-echelon vehicle routing problem and vaccine supply chain was presented to minimize the number of unsatisfied doses undelivered to customers. Cai et al. [40] proposed a decomposition-based multi-objective multiform evolutionary algorithm to solve multi-objective vehicle routing problems with time windows. Li et al. [41] proposed a multi-objective evolutionary algorithm based on decomposition with a customized replacement neighborhood and dynamic resource allocation for a multi-objective split delivery heterogeneous vehicle routing problem with three-dimensional loading. Pilati and Tronconi [42] addressed the optimization of the few-to-many pick-up and delivery vehicle routing problem by developing a multi-objective simulated annealing algorithm distinguished by four tailored local search operators. Each of these contributions illustrates the field’s shift toward addressing the nuanced challenges of logistics scheduling through the lens of multi-objective optimization, recognizing the indispensable role of economic, environmental, and social considerations in crafting sustainable, efficient, and fair vehicle routing solutions. A comparison of the existing works is given in Table 1. As shown in Table 1, [25] achieved the optimized result in which the equitable workload is assigned for each vehicle, while in [36], the service waiting time was considered. To the best of our knowledge, none of those or other unlisted studies considered both the total wait time of all points and the workload balance of vehicles.

While a significant array of multi-objective models and algorithms have been developed in recent research, most existing solutions for the cumulative capacitated vehicle routing problem (CCVRP) focus on singular objectives. This trend overlooks the multifaceted challenges inherent in real-world vehicle scheduling scenarios. Beyond the primary goal of rapid material delivery, considerations such as driver workload, vehicle workload balance, and operation times within safe limits are crucial for ensuring efficient and secure deliveries [25]. At the same time, the on-site commander needs to select the logistics transportation scheme according to the actual situation and emergency experience, that is, the algorithm needs to be able to output multi-objective-optimized transportation scheduling schemes for on-site decision-making. Given the sparse integration of CCVRP with multi-objective optimization, this paper introduces a novel approach, the multi-objective cumulative capacitated vehicle routing problem considering operation time (MO-CCVRP-OT). This model incorporates both the travel time of vehicles and the unloading time at disaster sites. Unloading times are pivotal, as they extend the operational duration of vehicles and, consequently, the waiting period for subsequent destinations, thus representing an element that cannot be disregarded. The MO-CCVRP-OT model identifies the following two optimization objectives: the minimization of the total wait time at disaster sites and a reduction in excess operation time costs for rescue vehicles. These objectives are chosen because they directly impact the two most concerned parties in disaster relief scenarios—the drivers, whose labor intensity is a key factor, and the victims, whose needs and interests are paramount. In essence, the MO-CCVRP-OT aligns more closely with the realities of emergency logistic by addressing both the logistical efficiency and the human element within disaster relief. 

CCVRP has been proven to be NP-hard [5]. MO-CCVRP-OT intensifies the complexity due to the need to balance multiple, often conflicting, objectives. To address CCVRP, various algorithms have been developed. Exact solution algorithms designed specifically for problem models are used to solve small instances [12]. Heuristic algorithms, especially meta-heuristic algorithms, can be adapted to overcome the computational complexity of exact solution methods and yields solutions for larger instances in a reasonable time span [9]. Wang et al. [43] proposed a disturbance-based local search algorithm to solve the multi-depot CCVRP, integrating disturbance operators and six local search operators to improve the exploration ability. Ngueveu et al. [5] adopted the memetic algorithm, combined with the heuristic cost change calculation method, to solve the CCVRP. Wang et al. [44] proposed an ant colony algorithm based on a hybrid saving algorithm and a simple two-stage 2-opt algorithm to solve the multi-depot CCVRP. Pei et al. [13] used the improved max-min ant colony system algorithm to solve the cumulative heterogeneous fleet emergency vehicle routing problem with demand weighting. However, these algorithms, geared toward single-objective optimization, fall short in addressing MO-CCVRP-OT’s multifaceted requirements. Multi-objective problems demand solutions that account for several objectives simultaneously, often necessitating algorithms that yield multiple non-dominant solutions forming a Pareto front. To this end, Gragnaniello et al. [45] explored brute-force multi-objective optimization for thermal management applications, while Bianco et al. [46] leveraged a genetic algorithm for optimizing a heat recovery and ventilation unit’s design, exemplifying the utility of multi-objective optimization in yielding non-dominated solutions across varied domains [25,31,33,36,37,38]. Particle swarm optimization has been prominently featured in solving multi-objective issues, including diverse applications, from vehicle routing problems [29] to wind turbine optimization [47]. Despite their advantages, genetic algorithms often face slow convergence, and particle swarm optimization may encounter local optimization pitfalls. The grey wolf optimizer (GWO) [48], introduced in 2014, stands out by simulating grey wolves’ social hierarchy and hunting strategies, offering rapid convergence and precise solutions to challenges ranging from face image segmentation [49] and the optimal reference tracking control problem [50] to brain tumor detection [51] and short-term power load forecasting [52].

In response to the complex requirements of multi-objective logistics optimization problems, this paper presents the novel dynamic grey wolf optimizer algorithm with floating 2-opt (DGWO-F2OPT). This innovation is a multi-objective adaptation of the original grey wolf optimization algorithm designed to tackle these intricate problems effectively. The algorithm uses real number encoding and decodes using the method of combining the bisection random key and ROV rule. To enhance its optimization capabilities, the algorithm introduces a dynamic non-dominated solution set update strategy, which includes a temporary storage mechanism for non-dominated solutions obtained during the solving process. Also incorporated is a wolf leader selection mechanism that randomly generates elements from the dynamic non-dominated solution set. Moreover, a multi-objective improved 2-opt local search strategy for MO-CCVRP-OT is proposed to effectively solve MO-CCVRP-OT by submitting the convergence speed of the algorithm. Finally, the effectiveness of the proposed DGWO-F2OPT algorithm is proved by two multi-objective problem-solving experiments.

The main contribution of this work can be summarized as follows: (1) Aiming to minimize total wait time at disaster sites and reduce excess operation time costs for rescue vehicles, this paper proposes a multi-objective model of MO-CCVRP-OT, which considers both the total wait times of all points and the workload balance of vehicles. (2) A dynamic grey wolf optimizer algorithm with floating 2-opt based on the GWO (DGWO-F2OPT) is proposed to solve the MO-CCVRP-OT, which designs a dynamic non-dominated solution set update strategy and a wolf leader random selection mechanism. A multi-objective improved floating 2-opt local search strategy is proposed to enhance the local searchability. (3) Four experiments are designed to verify the effectiveness of the proposed DGWO-F2OPT and its improved strategies. 

The remainder of this paper is organized as follows:

Section 2 describes the MO-CCVRP-OT model. Section 3 introduces a dynamic grey wolf optimizer algorithm with floating 2-opt to solve the MO-CCVRP-OT model. Section 4 presents the experimental results and a case study on the real rescue case of 1999 Chi-Chi earthquake to evaluate the DGWO-F2OPT. Section 5 concludes the paper and presents future work directions.

## 2. Problem Description and Mathematical Model

**Figure 1:**
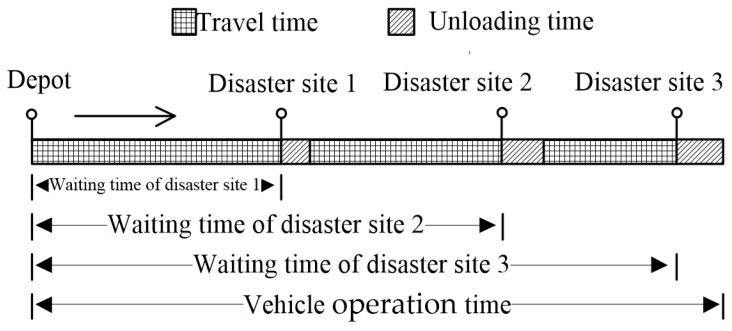
Diagram of customer wait time and vehicle operation time.

The essence of the MO-CCVRP-OT is described in the following scenario: There is one depot and multiple vehicles. Each vehicle needs to serve multiple disaster-affected points. The vehicles depart from the depot, drive to the first disaster-affected point, perform unloading operations, drive from the first point to the second point, perform unloading operations again, and continue to follow this pattern until they reach the final disaster-affected point and complete the unloading operations there. The major concern for the disaster-affected points is the wait time prior to the arrival of the vehicles, with the hope that the vehicles will arrive as quickly as possible. Additionally, the problem necessitates the consideration of the operation time for each vehicle, which consists of both travel and unloading times. To systematically manage this, the MO-CCVRP-OT establishes a “rated operation time” and a “maximum operation time” for vehicles. If the operation time of a vehicle exceeds the rated operation time, additional expenditures will be required, while the maximum operation time represents an absolute limit that cannot be surpassed. Assuming a certain vehicle is scheduled for delivery at three disaster-affected points, the wait time and vehicle operation time at the disaster-affected points are shown in Figure 1.

In order to enhance the precision and reliability of the model, several key assumptions have been established as follows:

1. Disaster Response Scope: The model is designed with the intent of comprehensively addressing the immediate impact of an initial disaster across multiple points. The objective is to optimize the distribution routes to ensure that essential supplies reach all affected locations promptly. We disregard potential damage from aftershocks or secondary disasters, as well as the possibility of transportation failures due to the initial or secondary disaster;
2. Wait Time Consideration: The elapsed time from when a disaster occurs to the moment a relief vehicle (or truck) arrives at an affected point is referred to as the “wait time” of that point. To assess the overall effectiveness of a relief strategy, the wait times of all points (or all locations affected by the disaster) are added up. The model does not factor in the transportation priority that might arise from emergency triage considerations or the scale of the affected population at each site;
3. Deterministic Model Parameters: The proposed model operates on a deterministic basis, meaning that the affected population and the so-called “fuzzy” demand—or uncertain and variable demand—at the disaster-affected sites are assessed and estimated in advance of the optimization process;
4. Focus on Timeliness and Safety: In emergency rescue scenarios, timeliness requirements and transportation safety are prioritized [53]. Fuel consumption or environmental impacts like CO2 emission are ignored. However, the maximum operation time of the vehicles is considered, analogous to limiting their fuel capacity;
5. Definition of Operation Time: For each vehicle, the operational time spans from the moment it departs from the depot until it reaches the final disaster-affected point on its itinerary. This encompasses the entirety of the route that the vehicle travels while carrying out its rescue assignment.

In MO-CCVRP-OT, the following conditions need to be satisfied: Each disaster-affected point has a certain demand for rescue materials, and only one vehicle visits each point. The number of vehicles scheduled is less than or equal to *V*, and all vehicles depart from the depot. The total transportation demand of the disaster-affected points shall not exceed the upper limit of the vehicle carrying capacity, and the actual operation time of each vehicle shall be less than the maximum operation time. There is no capacity limit for the depot. There are two objectives. One is to minimize the cumulative wait time of all disaster-affected points, and the other is to minimize the cost due to rescue vehicles exceeding the operation time.

The mathematical variables are defined as shown in Table 2.

The mathematical model for MO-CCVRP-OT relies on formulating and optimizing two different objective functions. These functions are designed to quantify and minimize the key goals of the problem, as follows:

(1) $$\min\;Z_{1}=\sum_{k=1}^{V}\sum_{i=1}^{n}TCUM_{i,k},$$

(2) $$\min\;Z_{2}=\sum_{k=1}^{V}FOPOver\times \max(TAct_{k}-TNormal,0)\;,$$

where Equation (1) represents the minimum wait time of vehicles at all disaster-affected points, and Equation (2) represents the minimum cost incurred by the excess operation time of rescue vehicles, which is nonlinear. Obviously, MO-CCVRP-OT is a nonlinear integer programming problem.

When it comes to the delivery of relief supplies post-disaster, the focus tends to shift away from cost-effectiveness toward more critical factors like promptness and the reliability of transport. Timeliness is primarily measured by the wait time for the vehicles to reach all designated points, an aspect that directly reflects the urgency felt by disaster victims awaiting assistance. Equally important is the workload of the drivers to ensure they operate within a reasonable timeframe without excessive fatigue, which may lead to increased wait times at affected locations. This juxtaposition—the wait time for aid versus the operational time of rescue vehicles—embodies the competing interests of the victims and the logistical staff. By prioritizing these twin goals, our approach acknowledges the practicality of addressing both the immediate needs of those impacted by the disaster and the well-being of the transportation team members who are instrumental in the relief efforts.

In the context of our problem, all vehicles begin their journey from a central point—the depot—and from there, they proceed to their respective assigned disaster-affected points. Each disaster-affected point is visited by exactly one vehicle, as shown in Equation (3). This constraint is applied only to the disaster point set, excluding the depot.

(3) $$\sum_{k=1}^{V}b_{i,k}=1,\;i\in P\;,$$

As assumed in our problem, the number of vehicles scheduled is less than or equal to *V*. From the perspective of the depot, Equations (4) and (5) stipulate that the number of vehicles departing from or arriving at the depot does not exceed the maximum allowable number of vehicles.

(4) $$\sum_{j=1}^{n}a_{1,j,k}\leq V,$$

(5) $$\sum_{j=1}^{n}a_{j,1,k}\leq V,$$

For each vehicle assigned, the number of times it arrives at, departs from, and visits each disaster point stays consistent throughout the operation, as show in Equation (6).

(6) $$\sum_{j=1}^{n}a_{i,j,k}=\sum_{j^{\prime }=1}^{n}a_{j^{\prime },i,k}=b_{i,k},\;i\in P,k=1,\ldots ,V\;,$$

As mentioned above, the total demand of the disaster-affected points allocated for a vehicle does not exceed the upper limit of the vehicle’s carrying capacity. We have the following constraint:

(7) $$\sum_{i=1}^{n}q_{i}b_{i,k}\leq U,\;k=1,\ldots ,V\;.$$

If the operation time of a vehicle exceeds the rated operation time, additional expenditures will be incurred, and the maximum operation time cannot be exceeded. We have the following constraint:

(8) $$TCUM_{i,k}+y_{i}+\frac{d_{i,j}}{s}-(1-a_{i,j,k})M\leq TCUM_{j,k},\;\;i\in N,\;j\in N,\;k\in \{1,\ldots ,V\}\;,$$

(9) $$TAct_{k}=\underset{i\in N}{\max}(TCUM_{i,k}),\;\;k\in \{1,\ldots ,V\}\;,$$

(10) $$TAct_{k}\leq TOPMax,\;\;k\in \{1,\ldots ,V\}\;,$$

where, in Equation (8), *M* is a large integer, which is used to avoid the sub-tour and calculate the wait time for each point at the vehicle’s arrival. Equation (9) obtains the actual operation time of each vehicle, while Equation (10) indicates that the actual operation time of each vehicle cannot exceed the maximum operation time.

In this problem, three decision variables are introduced as follows:

(11) $$TCUM_{i,k}\geq 0,\;i\in N,\;k\in \{1,\ldots ,V\}\;,$$

(12) $$b_{i,k}\in \{0,1\},\;i\in N,k=1,\ldots ,V\;,$$

(13) $$a_{i,j,k}\in \{0,1\},\;i,j\in N,k=1,\ldots ,V\;,$$

where Equation (11) defines the numerical type of *T*_cum*,i,k*_, while Equations (12) and (13) define the 0–1 integers of decision variables *a_i,j,k_* and *b_i,k_*. In Equation (12), for the depot, i.e., *i* = 1, b*_i_*_,*k*_ will be 0, as the values will not be set in Equations (3) and (6).

In Equation (8), when $a_{i,j,k}=0$, the inequality holds because the left part of the inequality is a large negative integer, which means that $TCUM_{j,k}$ is not dependent on $TCUM_{i,k}$ when vehicle *k* does not travel from *i* to *j*. If $a_{i,j,k}=1$, then $(1-a_{i,j,k})M=0$. In this case, compared to $TCUM_{i,k}$, $TCUM_{j,k}$ increases in travel time from *i* to *j* and the service time of *i* when vehicle *k* travels from *i* to *j*. Suppose that sub-tour (*i*, *j*, *l*, i^*^) exists in the solution; *i* and i^*^ are the same customer. $TCUM_{i^{*},k}$ will be added up travel time from *i*, *j*, *l*, to i^*^ and a service time of *i*, *j*, and *l.* $TCUM_{i^{*},k}$ is not equal to $TCUM_{i,k}$; it does not hold since *i* and i^*^ are the same customer. Thus, Equation (8) can be used to avoid sub-tour.

To sum up, the constraints and other assumptions we considered are shown in Table 3.

## 3. Design of the Dynamic Grey Wolf Optimizer Algorithm with Floating 2-opt

The dynamic grey wolf optimizer algorithm with floating 2-opt (DGWO-F2OPT) defines *PopSize* as the size of the grey wolf pack, *MaxIter* as the maximum number of iterations, and the position vector as *m*-dimension. The difference between multi-objective optimization GWO and traditional GWO lies in the evaluation mechanism of individual grey wolves and the generation mechanism of leader wolves.

### 3.1. Encoding Method and Decoding Strategy

The encoding of the grey wolf position vector in this paper adopts an *m*-dimensional real number vector, that is, *X* = (*x_i_*, *x_2_*, …, *x_i_*, …, *x_m_*), and $x_{i}\in [LB,UB]$, the lower and upper bounds, respectively. This paper adopts a combination of equal-division random keys [54] and ROV rules [55] for decoding.

The first step is to allocate *m* components of the position vector *X* to *K* vehicles using the equal-division random key method. Firstly, the interval [*LB*,*UB*] is divided into *K* intervals, that is, add *K* − 1 segmentation points in the interval [*LB*,*UB*], and the *i*-th segmentation point *DP_i_* = $i\times \frac{UB-LB}{K},i\in \{1\ldots K-1\}$. After the segmentation, *K* intervals are formed, which are [*LB*, *DP*_1_], …, [*DP_i-_*_1_, *DP_i_*], …, [*DP_K_*_-1_, *UP*]. Then, *m* components are allocated according to *K* intervals to form *K* subsets.

The second step is to encode the *K* subsets respectively using ROV rules. *K* integer sequences are obtained, which represent *K* vehicle paths. Then, 1 is added to each component of *K* integer sequences, and the union of all components in all integer sequences is the set of disaster-affected points {2, …, *n*}.

### 3.2. Multi-Objective Evaluation Mechanism for Grey Wolf Individuals

In contrast to single-objective algorithms, the double-objective optimization problem presented in this paper yields two fitness values for each evaluated grey wolf individual. Then, $FIT_{i,1}$ represents the cumulative wait time at all disaster-affected locations within the solution attributed to grey wolf individual *i*. Meanwhile, $FIT_{i,2}$ quantifies the total extra costs incurred due to the extended operation time of all vehicles within the same solution. In MO-CCVRP-OT, achieving lower values for both $FIT_{i,1}$ and $FIT_{i,2}$ is preferable. Similarly, Two fitness values for grey wolf individual *j* are denoted as $FIT_{j,1}$ and $FIT_{j,2}$. 

Comparing grey wolf individuals *i* and *j*, if either $FIT_{i,1}$ ≤ $FIT_{j,1}$ and $FIT_{i,2}$ < $FIT_{j,2}$, or $FIT_{i,1}$ < $FIT_{j,1}$ and $FIT_{i,2}$ ≤ $FIT_{j,2}$, the solution of grey wolf *j* is considered to be dominated by that of grey wolf *i*, denoted as i≺j. If the solution of grey wolf *i* is not dominated by the solution of any other individual, then the solution of grey wolf *i* is called a non-dominated solution.

### 3.3. Dynamic Non-Dominated Solution Set Update Strategy

Grey wolves are divided into the following four levels: *α*, *β*, *δ*, and *ω*. The three grey wolf individuals (*α*, *β*, *δ*) with the best fitness in the traditional grey wolf optimizer algorithm are relatively constant. When an ordinary grey wolf is superior to *α*, *β*, or *δ*, the leader wolf will be replaced. However, DGWO-F2OPT adopts a dynamic non-dominated solution set update strategy, which dynamically saves the non-dominated solutions obtained during the solving process and randomly selects the leader wolf from the dynamic non-dominated solution set for updating the position of the grey wolf. Parameter *MP* is defined as the maximum number of dominated solutions in the dynamic non-dominated solution set. Specifically, two sets are used in the dynamic non-dominated solution set update strategy. One is the fitness set for the non-dominated solution set with elements (*fitness*_1_, *fitness*_2_). The other is the *m*-dimension position set for the non-dominated solution set with elements of position vectors (*x*_1_, *x*_2_, …, *x_i_*, …, *x_m_*). The elements of the two sets have a one-to-one correspondence relationship. Both sets are empty at initialization.

During algorithm iteration, the new position obtained by updating each grey wolf is decoded to obtain the solution, and two fitness values for the current grey wolf are obtained through a multi-objective evaluation. The fitness set for the non-dominated solution set is traversed for comparison, and the dominant relationship between the solution of the current grey wolf and the solutions in the non-dominated solution set is determined. In the steps below, *i* is the current grey wolf, and $Num_{\mathrm{nd}}$ is the number of the non-dominated solution set. *FitnessSet* is the fitness set for the non-dominated solution set, *PosVecSet* is the position set for the non-dominated solution set, and both have $Num_{\mathrm{nd}}$ elements as follows:

1. If $j≺i,\exists j\in [1..Num_{\mathrm{nd}}]$, *FitnessSet* and *PosVecSet* will not be updated, and the traversal will stop;
2. If $i≺j,\exists j\in [1..Num_{\mathrm{nd}}]$, the non-dominated solution *j* will be replaced by *i* as follows: the *j*th elements in *FitnessSet* and *PosVecSet* will be updated with the fitness and the position vectors of *i*; the traversal will continue to compare subsequent non-dominated solutions. If other non-dominated solutions are dominated by *i*, the relevant elements in the fitness set and position set of other non-dominated solutions will be deleted;
3. If the traversal process is completed, and the solution represented by *i* is neither dominated by any solution within the non-dominated solution set nor does it dominate any of those solutions, then *i* will be considered a non-dominated solution and subsequently added to the non-dominated solution set. Consequently, the fitness of *i* will be added to *FitnessSet* as a new element, and the positional vector of *i* will be incorporated into *PosVecSet*;
4. If the number of elements in both *FitnessSet* and *PosVecSet* exceeds *MP*, the original element exit operation will start as follows: according to the fitness values of the first dimension of the elements in *FitnessSet*, the interval from the minimum value to the maximum value will be equally divided into 10 sub-intervals with the same range. The number of elements in each of these sub-intervals will be calculated. A solution will be selected randomly from the sub-interval with the largest number of elements, and the elements in *FitnessSet* and *PosVecSet* will be deleted accordingly.

### 3.4. Grey Wolf Operation Update

The principle and detailed process of the GWO algorithm can be found in reference [48,56]. In the GWO algorithm, *it* and *MaxIter* represent the current number of iterations and the maximum number of iterations, respectively. The control vector is *wa* = 2− 2(*it*/*MaxIter*). The synergy coefficients (*WC_1_*, *WC_2_*, *WC_3_*) are calculated as shown in Equation (14), and *WA_1_*, *WA_2_*, and *WA_3_* are calculated as shown in Equation (15). The specific equation update for the position of the *ω*-level grey wolf *X*(*it*) is shown in Equations (14) to (22) as follows:

(14) $$WC=2r_{1}\;,$$

(15) $$WA=2wa\times r_{2}-wa\;,$$

(16) $$WD_{\alpha }=|WC_{1}\cdot X_{\alpha }(it)-X(it)|,\;$$

(17) $$WD_{\beta }=|WC_{2}\cdot X_{\beta }(it)-X(it)|\;,$$

(18) $$WD_{\delta }=|WC_{3}\cdot X_{\delta }(it)-X(it)|\;,$$

(19) $$X_{1}=X_{\alpha }-WA_{1}WD_{\alpha }\;,$$

(20) $$X_{2}=X_{\beta }-WA_{2}WD_{\beta }\;,$$

(21) $$X_{3}=X_{\delta }-WA_{3}WD_{\delta }\;,$$

(22) $$X(it+1)=(X_{1}+X_{2}+X_{3})/3\;,$$

where, *X_α_*(*it*), *X_β_*(*it*), and *X_δ_*(*it*) represent the current positions of leader wolf *α*, *β*, and *δ*, respectively; *X*(*it*) and *X*(*it* + 1) represent the current position and the updated position of the *ω* wolf, respectively; and *r*_1_ and *r*_2_ are random numbers in the range [0, 1].

### 3.5. Fitness Function

According to the problem definition in Section 1, the corresponding fitness function is used in the algorithm for evaluating the grey wolf pack and selecting non-dominated individuals. In this paper, the fitness function is as follows:

(23) $$fitness_{1}=\sum_{k=1}^{V}\sum_{i\in P}TCUM_{i,k}+M\sum_{k=1}^{V}\max(\sum_{i\in P}\left(b_{i,k}q_{i}\right)-U,0),$$

(24) $$fitness_{2}=\sum_{k=1}^{V}FOPOver\times \max(TAct_{k}-TNormal,0)+M\sum_{k=1}^{V}\max(TAct_{k}-TOPMax,0)+M\sum_{k=1}^{V}\max(\sum_{i\in P}\left(b_{i,k}q_{i}\right)-U,0),$$

where *M* is a large integer. The fitness function (*fitness*_1_) is calculated in two parts. The first part represents the sum of the wait time at all disaster-affected points, and the second part represents the penalty for the actual vehicle load exceeding its maximum carrying capacity. The fitness function (*fitness*_2_) is calculated in three parts. The first part represents the additional cost incurred by the excess operation time of the vehicle beyond its rated operation time. The second part represents the penalty for the operation time of the vehicle exceeding the maximum operation time. The third part represents the penalty for the actual vehicle load exceeding its maximum carrying capacity. When the actual vehicle load exceeds the maximum carrying capacity, the maximum value should be taken for both *fitness*_1_ and *fitness*_2_. When the vehicle operation time exceeds the maximum operation time, the maximum value should only be taken for *fitness*_2_.

### 3.6. Improved Multi-Objective Local Search Strategy

In order to improve the local search capability of DGWO-F2OPT, this paper proposes a floating 2-opt (F2Opt) local search strategy. Fundamentally, F2Opt is an enhancement of the conventional 2-opt strategy. The 2-opt local search modifies the existing solution by eliminating two edges in a given loop and introduces two new edges connected to the four vertices of the removed edges, ultimately aimed at reducing the total cost. Since MO-CCVRP-OT is a multi-objective optimization problem, and one solution involves two fitness values, the change in overhead cannot be determined directly. Therefore, for MO-CCVRP-OT, the F2Opt strategy also performs the same 2-opt operation. The difference lies in calculating the overhead using a comprehensive fitness. In this regard, two evenly-distributed random numbers within the (0..1) interval, denoted as *ro*_1_ and *ro*_2_ (where *ro*_1_ + *ro*_2_ = 1), are generated. The comprehensive fitness *CF* is then computed according to Equation (25).

(25) $$CF=ro_{1}\sum_{i\in P^{\prime }}TCUM_{i,k}+ro_{2}\times FOPOver\times \max(TAct_{k}-TNormal,0),$$

where, $P^{\prime }$ is a subset of *P*, and the set of nodes accessed by vehicle *k* is expressed as *k* ∈ [1..*V*]. Two conditions need to be satisfied to determine the transformation of the two edges as follows:

1. After the two edges are transformed, the comprehensive fitness of all disaster-affected points on the path will decrease;
2. In the path obtained by transforming two edges, the total vehicle operation time upon delivery shall not exceed *TOPMax*.

In order to simplify the calculation of *CF*, the wait time of node *i* on the path of vehicle *k* (*DN_ik_*) is precalculated, and *r_i_* is defined as the path position of node *i*. The first node is *r*_1_ = 0, and it increases thereafter. Node *i* is located on path group *k*, and we define a weighted action coefficient *AF_i_* = max(|*G_k_*|-2-*r_i_*, 0) for node *i* on the path *G_k_* of vehicle *k*, which is the number of disaster-affected points after node *i* in the path group. The calculation of overhead change can be divided into three parts. The first part is the variation value of the vehicle path and distance change after considering the weighted action coefficient. The second part is the variation value of the unloading time at each disaster-affected point after considering the weighted action coefficient. The third part is the variation value of the vehicle’s completion time at the last node before and after the change.

### 3.7. Algorithm Steps

The specific steps for the DGWO-F2OPT algorithm for solving MO-CCVRP-OT are listed below, with the termination condition of the algorithm being the number of iterations reaching the set maximum number. The specific steps are as follows:

Step 1: The fitness set and position set for the non-dominated solution set are initialized. The grey wolf pack $X=\{x_{1},x_{2},\ldots x_{i},\ldots ,x_{m}\},i\in \{1,2,\ldots ,PopSize\}\;,$ is generated randomly with *it* = 0;

Step 2: A path is decoded and generated for the position vector of each grey wolf in X according to Section 3.1;

Step 3: The path is checked to see if it meets the load constraint of Equation (6). If it does, a floating 2-opt local search strategy will be applied for the path according to Section 3.6, and the algorithm will proceed to the next step. If the load constraint is violated, both *fitness*_1_ and *fitness*_2_ will be set to *M*, and the algorithm will skip to Step 5;

Step 4: The fitness of the individual grey wolf is calculated. The dynamic non-dominated solution set update strategy will be executed, and the algorithm will move to the next step;

Step 5: From the position set for the non-dominated solution set, the 3 position vectors *α*, *β*, and *δ* are randomly selected;

Step 6: The grey wolf update operation is performed on grey wolf pack *X*;

Step 7: Let *it* = *it* + 1, and if *it* ≥ *MaxIter*, the algorithm will move to the next step. Otherwise, it will skip to Step 2;

Step 8: The algorithm will output the paths represented by all individuals in the dynamic non-dominated solution set.

## 4. Experiments and Analysis

### 4.1. Experiment Instances and Environment

To accurately analyze the effectiveness of DGWO-F2OPT in solving MO-CCVRP-OT, a CVRP standard calculation example was used and modified. Node information for the experiment example is shown in Table 4. Node 1 represents the depot, and nodes 2–40 represent disaster-affected points.

In addition, this experiment was conducted on a personal computer with a Windows 10 64-bit operating system, an Intel (R) Core (TM) i5-9400 CPU @ 2.90 GHz processor, and 16 GB of RAM. The simulation computing environment used is MATLAB 2017a (9.2).

### 4.2. Experiment Results and Analysis

Experiment 1: This experiment verifies the effectiveness of DGWO-F2OPT in solving MO-CCVRP-OT. In this experiment, *MaxIter* = 4000, *PopSize* = 40, and *MP* = 80. The maximum number of available vehicles is *V* = 6, vehicle capacity limit *U* = 140 (hundredweight, cwt), vehicle operating speed *s* = 1 (km/m), rated operation time of vehicle *TNormal* = 120 (min), additional expenditure per unit time *FOPOver* = 20 (Subsidy unit /m), and the maximum operation time *TOPMax* = 240 (min).

The algorithm was run 20 times, and the number of output non-dominated solutions and the run time were counted for each run. The results of the 20 runs are shown in Table 5. Column 1 in Table 5 is the sequence number for each run, Column 2 is the number of non-dominated solutions in the result for each run, and Column 3 is the running time of the algorithm for each run.

From the results in Table 5, the following can be seen:

1. The maximum number of non-dominated solutions is six, and the minimum value is one, indicating that the algorithm is effective in solving dual-constraint (load and operation time constraints) problems;
2. The maximum running time is 28.4 s, while the minimum is 15.821 s. The difference in each run is due to the fact that local optimization will only be performed in the algorithm when it meets the load constraint. The number of times the local search strategy is run varies due to the randomness of the algorithm.

The solution result with the sequence run number of four was analyzed. It outputs the most non-dominated solutions, that is, six non-dominated solutions, as shown in Table 6. Among the six non-dominated solutions the sequence number of one is the utopia optimum. Its *fitness*_1_ is 2797 and *fitness*_2_ is 2540.

**Figure 2:**
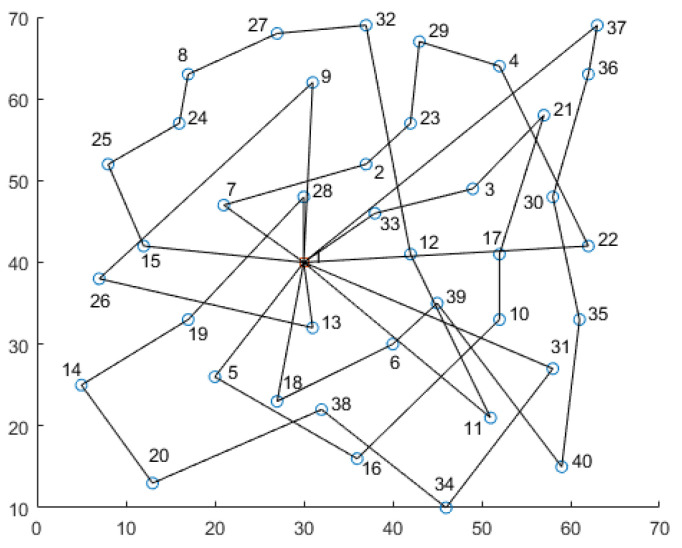
The solution paths for utopia optimum.

The solution path for the utopia optimum is shown in Figure 2, and the result information is shown in Table 7. In Figure 2, the blue circles are the disaster-affected points, and the red square is the depot. The numbers in Figure 2 represent the sequence number of the point according to Table 4. Compared to the regular solution path of CVRP, the lengths of all paths are relatively even in Figure 2 to balance the operation time of all vehicles. As shown in Figure 2, vehicles tend to visit the nearer points preferentially to shorten the wait time of all disaster-affected points. In Table 7, the column for the total load capacity represents the vehicle load at the time of departure, which is equal to the sum of demands required by all disaster-affected points on the path. The column for the wait time represents the sum of the wait time of all disaster-affected points at the arrival of vehicles on the path. The column for the operation time represents the vehicle operation time from departure to task completion at the last disaster-affected point on the path, and the exceeding column represents the portion of the vehicle’s operation time exceeding the rated operation time *TNormal*.

From Table 7 and Figure 2, the following can be seen:

1. In the utopia optimum solution, six vehicles are used. The total load capacities of vehicles on the six paths are 139, 115, 108, 108, 68, and 80, respectively, all of which are within the capacity limit of 140, meeting the number of vehicles constraint in Equation (4) and the load capacity constraint in Equation (6). The total load capacity of the six paths is equal to the total delivery demand required by the disaster-affected points, meeting the needs of these points;
2. For the solution paths of the utopia optimum solution, each vehicle will visit at least one disaster-affected point. Each disaster-affected point belongs to, and only belongs to, one simple loop, with each loop starting from and ending at the depot, meeting the constraints between the vehicle, path, and disaster-affected point in Equations (3) and (5). The operation times of vehicles on the six paths are 163, 139, 150, 155, 101, and 94, respectively, all of which are within the maximum operation time of 240. There are four vehicles that exceed the rated operation time of 120, which results in additional expenditures. The total overtime amount is 127, and the additional expenditure for overtime is 2540.
3. In the utopia optimum solution, the wait times of each disaster-affected point on the six paths are 577, 493, 593, 689, 308, and 137, respectively, for a total of 2797. The sums of the vehicle arrival times on all paths are relatively even, indicating that the output solution is high quality.

In summary, the dynamic grey wolf optimizer algorithm with floating 2-opt effectively obtained the non-dominated solution for MO-CCVRP-OT. Different solutions can be selected from the output results, such as solutions focusing more on the wait time of disaster-affected points or more on the overtime operation of vehicles. Compared with single-objective algorithms, it has significant advantages and can better support real-time decision-making for scheduling transportation in emergency logistics.

Experiment 2: In order to further analyze the solving capability of DGWO-F2OPT and the effectiveness of related strategies, this algorithm will be compared with two algorithms. One is the dynamic grey wolf optimizer algorithm with a traditional 2-opt local search strategy, labeled DGWO-2OPT. The DGWO-2OPT is similar to the DGWO-F2OPT but lacks the floating calculation and only adopts the ordinary 2-opt local search strategy, using the increase or decrease in cumulated wait time as the basis for calculating the expenditure difference before and after path changes. The other one to compare with is the dynamic grey wolf optimizer algorithm without any local search strategy, labeled DGWO.

The parameters are the same as in Experiment 1. The DGWO-2OPT algorithm and the DGWO algorithm were both run 20 times, and the number of output non-dominated solutions and run time were counted for each calculation. Invalid solutions among the output non-dominated solutions were eliminated before counting the number of output non-dominated solutions. The results of 20 calculations are shown in Table 8. 

In order to measure the quality of the output solutions, the result with the largest number of non-dominated solutions among 20 runs was selected for analysis. In Table 8, the solution result with the sequence run number of 14 was analyzed in the results of the DGWO-2OPT algorithm, as well as the sequence run number of 17 in the results of the DGWO algorithm. The sequence run number of four in the results of the DGWO-F2OPT algorithm in Table 5 was analyzed together. The averages of the two fitness values for the non-dominated output solutions were calculated. After calculation, the minimum average value of fitness for all non-dominated solutions was taken as the average value of fitness of the algorithm. The average values of fitness of the three algorithms are shown in Table 9. The average number of non-dominated solutions and average run times for the DGWO algorithm, the DGWO-2OPT algorithm, and the DGWO-F2OPT algorithm for 20 runs are also shown in Table 9. In Table 9, Column 2 is the average number of non-dominated solutions of 20 runs for the three algorithms. Column 3 is the average running time of 20 runs for the three algorithms. Column 4 is the average value of two fitness values of the utopia optimum solution for the three algorithms.

From the results in Table 8 and Table 9, the following can be seen:

1. The maximum number of non-dominated solutions solved by DGWO is 3, while the minimum is 1, and the average is 1.2. The maximum number of non-dominated solutions solved by DGWO-2OPT is 4, while the minimum is 1, and the average is 1.6. The average number of non-dominated solutions solved by DGWO-F2OPT is 2.2. DGWO-F2OPT obtained more non-dominated solutions than DGWO-2OPT and DGWO, while DGWO obtained the fewest non-dominated solutions. In terms of the number of non-dominated solutions, DGWO-F2OPT has a stronger solving capability than DGWO-2OPT and DGWO;
2. DGWO has a maximum run time of 6.976, a minimum run time of 6.618, and an average run time of 6.796. By contrast, DGWO-2OPT has a maximum run time of 19.894, a minimum run time of 15.536, and an average run time of 17.467. The average run time of DGWO-F2OPT is 21.086. DGWO-2OPT has a shorter run time than DGWO-F2OPT, while DGWO has the shortest run time. The major reason for the difference in computation times is due to the larger number of non-dominated solutions obtained by DGWO-F2OPT and the higher number of local optimizations, which results in a longer computation time;
3. The average value of fitness of DGWO is 6089.5, while that of DGWO-2OPT and DGWO-F2OPT are respectively 2845.5 and 2668.5. Obviously, DGWO is much weaker than DGWO-2OPT and DGWO-F2OPT in terms of the accuracy of the algorithms. The average value of two fitness values of the utopia optimum solution of DGWO-F2OPT is 6.22% smaller than that of DGWO-2OPT.

**Figure 3:**
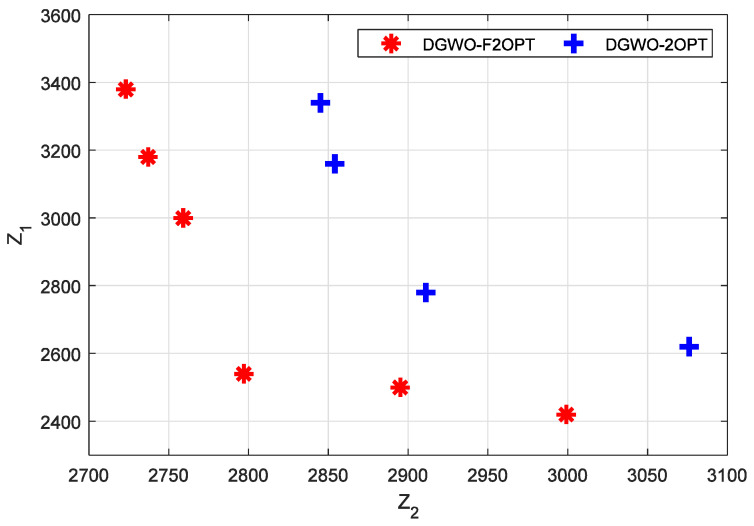
Result of non-dominated solutions of DGWO-F2OPT and DGWO-2OPT.

Due to the huge gap in the quality of the output between DGWO and the other two algorithms, we exclude DGWO in our further analysis. In other words, our further analysis was conducted on DGWO-2OPT and DGWO-F2OPT. Of the two algorithms, the running result that output the greatest number of non-dominated solutions was further analyzed. Among the non-dominated solutions obtained in a single run, the solutions whose fitness values contain penalties are considered invalid solutions and shall be eliminated. For DGWO-F2OPT, the solution result with the sequence number of four in Table 5 was taken for analysis. It contains six non-dominated solutions, which are all valid solutions. For DGWO-2OPT, the solution result with the sequence number 14 was taken for analysis. It contains four non-dominated solutions, which are all valid solutions. The distribution of the above 10 points is shown in Figure 3.

From Figure 3, it can be seen that the non-dominated solutions of DGWO-F2OPT tend toward the lower left corner compared with those of DGWO-2OPT, and the solutions of DGWO-2OPT tend toward the horizontal axis. The following can be concluded:

1. The non-dominated solutions of DGWO-F2OPT are of better quality than those of DGWO-2OPT. DGWO-F2OPT can obtain solutions with lower numerical values for both *Z*_1_ and *Z*_2_, while DGWO-2OPT can only obtain solutions with lower numerical values for *Z*_1_, resulting in the uneven distribution of solutions at the Pareto boundary. It can be concluded that the DGWO-F2OPT algorithm has a better search capability;
2. For the non-dominated solutions of DGWO-F2OPT, the utopia point in the bi-objective plane is (2723, 2420), and the utopia optimum is (2797, 2540). The utopia point and the utopia optimum for DGWO-2OPT are (2845, 2620) and (2911, 2780), respectively. The utopia point and the utopia optimum DGWO-F2OPT achieves have smaller values than those DGWO-F2OPT obtains. From the perspective of the utopia point and the utopia optimum of both algorithms, DGWO-F2OPT obtains higher quality solutions than DGWO-F2OPT;
3. The average value of the twelve finesses value of six solutions of DGWO-F2OPT is 2827.5, while that of the eight finesses value of four solutions of DGWO-2OPT is 2948.25. The average value of the finesses of DGWO-F2OPT is smaller than that of DGWO-2OPT. From the perspective of the average value of finesses of both algorithms, DGWO-F2OPT has a better solving ability.

**Figure 4:**
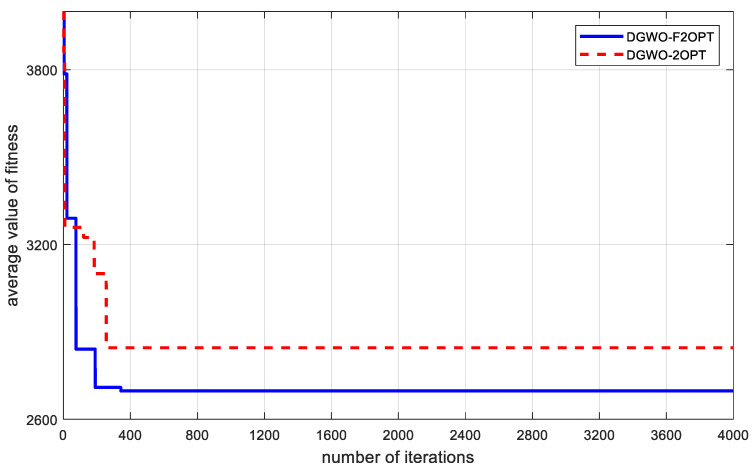
Change in average value of fitness with the number of iterations.

In order to measure the change in the level of fitness along with the number of iterations, the average of the two fitness values for non-dominated solutions is calculated in each iteration. After calculation, the minimum average value of fitness for all non-dominated solutions is taken as the average value of fitness of this iteration. In the above three solution results, the change in the fitness average, along with the number of iterations, is shown in Figure 4.

From Figure 4, the following can be seen:

1. The optimization outcomes achieved by DGWO-F2OPT, which incorporates the floating 2-opt local search strategy introduced in this study, are superior to those obtained with DGWO-2OPT. It is evident that the local search method employed here leverages a comprehensive fitness approach (for expenditure calculation based on wait time) that is aptly tailored for addressing MO-CCVRP-OT. This strategy not only enhances the global convergence potential of the algorithm but also bolsters the precision of the solutions it yields. Furthermore, the floating 2-opt component of the local search strategy contributes significantly to the algorithm’s refined local searching capabilities;
2. Since a multi-objective problem makes searching for a solution more difficult, we set the iteration number to 4000 to exhaust the solution finding. Results show that two algorithms reach convergence before 400 iterations. This implies that the floating 2-opt local search strategy improves the convergence speed to a great extent. Meanwhile, from the running time in Table 9, the average running times of DGWO-F2OPT and DGWO-2OPT are respectively 21.086s and 17.467s. The algorithms are acceptable regarding the solving time spent.

It can be seen that the model and solving algorithm proposed in this paper have profound significance and value in practical applications.

Experiment 3: In order to further analyze the solving capability and the suitability of the main algorithm of DGWO-F2OPT for our problem, this algorithm will be compared with an algorithm that is based on the neighborhood search algorithm with a greedy swap strategy and a 2-opt local search strategy [57], labeled NS-2OPT. NS-2OPT is also applied to solve MO-CCVRP-OT.

**Figure 5:**
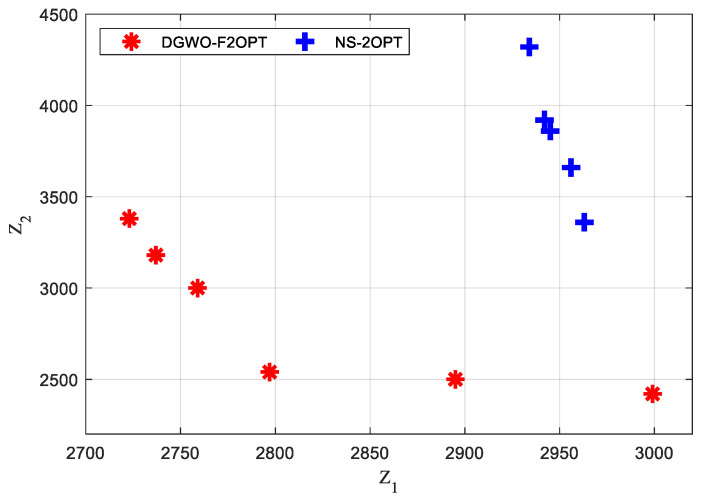
Result of non-dominated solutions of DGWO-F2OPT and NS-2OPT.

The problem parameters were the same as those in Experiment 1. The NS-2OPT algorithm was run 20 times, and the number of output non-dominated solutions and run time were counted for each calculation. The solutions of DGWO-F2OPT are adopted from Experiment 1. Invalid solutions among the output non-dominated solutions were eliminated before counting the number of output non-dominated solutions. Of DGWO-F2OPT and NS-2OPT, the maximum number of output non-dominated solutions of NS-2OPT is five, while that of DGWO-F2OPT is eight. The running result that output the greatest number of non-dominated solutions was further analyzed. The results of the two algorithms are shown in Figure 5. 

From Figure 5, it can be concluded that:

1. For the non-dominated solutions of DGWO-F2OPT, the utopia point in the bi-objective plane is (2723, 2420), and the utopia optimum is (2797, 2540). The utopia point and the utopia optimum for NS-2OPT are (2934, 3360) and (2963, 3360), respectively. The utopia point and the utopia optimum DGWO-F2OPT achieves have smaller values than those DGWO-F2OPT obtains. From the perspective of the utopia point and the utopia optimum of both algorithms, DGWO-F2OPT obtains higher-quality solutions than DGWO-F2OPT;
2. The average value of the 12 finesses value of six solutions of DGWO-F2OPT is 2827.5, while that of the 10 finesses value of four solutions of NS-2OPT is 3386. The average value of finesses of DGWO-F2OPT is 16.49% smaller than that of NS-2OPT. From the perspective of the average value of finesses of both algorithms, DGWO-F2OPT has better solving ability.

Experiment 4: To verify the effectiveness of MO-CCVRP-OT, a reference case from [58] is introduced, which is the real rescue case of the 1999 Chi-Chi earthquake in Taiwan. In order to make the case more realistic, the latitude, longitude, and total population are taken to model this real case as a MO-CCVRP-OT problem, which is solved using the proposed DGWO-F2OPT. As a comparison, according to this case, a capacitated vehicle routing problem considering operation time (CVRP-OT), which takes minimizing total traveling time as the single objective and considers the operation time, is constructed. The CVRP-OT model can be considered to be an instinctive emergency logistics model, taking the shortest total traveling time of all vehicles as the objective and considering the constraint of the maximum vehicle operation time. To solve CVRP-OT, the GWO-2OPT-SO algorithm is designed based on GWO, in which the encoding and decoding strategy mentioned in Section 3.1 is introduced, and the traditional 2-OPT local search strategy is adopted. Both algorithms run 20 times, and each obtains 20 results. In the results of MO-CCVRP-OT, the result with the lowest utopia point value is selected for analysis. In the results of CVRP-OT, the result that the minimum solution objective obtained is selected for analysis. Because there are several solutions in the result of MO-CCVRP-OT, the utopia optimum solution is selected among the solutions. Therefore, these two selected solutions are compared. The utopia point in the bi-objective plane is (773.62,534.4), The utopia optimum of MO-CCVRP-OT is (811.13, 535.2). The point for the solution of CVRP-OT is (819.78, 534.4). The comparison experiment results are shown in Table 10. 

The comparative analysis reveals that the total travel time for all vehicles is greater in the MO-CCVRP-OT model, while effectively reducing the accumulated wait time. Although the overall transportation cost is lower, the optimal total path length is not the primary consideration for emergency relief situations. Unlike the CVRP-OT model, the MO-CCVRP-OT model prioritizes minimizing the cumulative wait time at disaster sites. The importance of rescue time and the reliability of transportation are fully considered. The cumulative wait time for all the emergency materials was shortened to a better time, which was reduced by 1.06% compared with the CVRP model. The Euclidean distance from the Utopia point of MO-CCVRP-OT is 18.72% lower than that of CVRP-OT. The comparison experiment shows the practicability of the model and the effectiveness of the algorithm.

## 5. Conclusions

This paper proposes a multi-objective cumulative capacitated vehicle routing problem considering operation time. The model considers the unloading time of disaster relief materials at disaster-affected points. The two objectives for optimization are the cumulative wait time at all disaster-affected points upon vehicle arrival and the additional expenditure caused by the exceeded operation time of rescue vehicles. These considerations make the problem model more aligned with the actual requirements of emergency logistics. Based on the traditional grey wolf optimizer algorithm, this paper proposes a dynamic grey wolf optimizer algorithm with floating 2-opt to solve MO-CCVRP-OT. The algorithm employs a decoding strategy combining real number encoding and equally divided random keys with ROV rules. In addition, a dynamic non-dominated solution set update strategy is suggested that incorporates a temporary storage mechanism for the non-dominated solution set and a leader wolf generation mechanism based on this dynamic set. To solve MO-CCVRP-OT efficiently and increase the algorithm’s convergence speed, a multi-objective improved floating 2-opt (F2OPT) local search strategy is proposed. The utopia optimum solution of DGWO-F2OPT has an average value of two fitness values that is 6.22% lower than that of DGWO-2OPT. DGWO-F2OPT’s average fitness value in the algorithm comparison trials is 16.49% less than that of NS-2OPT. In the model comparison studies, MO-CCVRP-OT is 18.72% closer to the utopian point in Euclidean distance than CVRP-OT. Our next research endeavors can involve the following: (1) the model will be established with the consideration of the importance of catastrophe locations, vehicle fuel consumption, recurring material needs, and other emerging aspects. (2) Reinforcement learning will be engaged and used to improve our intelligent algorithm. (3) More local and neighborhood search techniques need to be researched and incorporated into our intelligent algorithm.

## Figures and Tables

**Table 1:** Comparison of studies on multi-objective CVRP in 2023–2024.

Research	Is Total Wait Time of All Point Considered	Is Workload Balance of Vehicles Considered
Wang et al. (2023) [25]	No	Yes
Amiri et al. (2023) [26]	No	No
Elgharably et al. (2023) [27]	No	No
Wang et al. (2023) [28]	No	No
Kuo et al. (2023) [29]	No	No
Comert et al. (2023) [30]	No	No
Wang et al. (2023) [31]	No	No
Liang et al. (2023) [32]	No	No
Menares et al. (2023) [33]	No	No
Soriano et al. (2023) [34]	No	No
Wang et al. (2023) [35]	No	No
Wang et al. (2023) [36]	Yes	No
Wang et al. (2023) [37]	No	No
Yin (2024) [38]	No	No
Al Theeb et al. (2024) [39]	No	No
Cai et al. (2024) [40]	No	No
Li et al. (2024) [41]	No	No
Pilati and Tronconi [42]	No	No
This Paper	Yes	Yes

**Table 2:** List of nomenclature.

Nomenclature	Meaning
Sets:	
*N*	Node set, N=P∪R
*P*	Disaster point set, *P* = {2, 3, …, *n*}
*R*	Depot set, Its value is {1}
Indices:	
*i*, *j*	Index of Node set
*k*	Index of Vehicles
Parameters:	
*n*	Number of nodes
*q_i_*	Transportation of disaster point *i*
*y_i_*	Unloading time in disaster point *i*
*d_i,j_*	Travel distance from *i* to *j*
*V*	Maximum number of vehicles available in the depot
intermediate variable	
*TAct_k_*	Actual operation time of each vehicle *k*
Constants:	
*s*	Travel speed of vehicles
*U*	Upper limit of vehicle load
*TNormal*	Rated operation time of vehicles
*FOPOver*	Additional expenditure per unit time for the excess part when the actual running time of the vehicle exceeds *TNormal*
*TOPMax*	Maximum operation time of vehicles
Decision variable:	
*a_i_*_,*j*,*k*_	1 when vehicle *k* travel from node *i* to *j*, otherwise 0
*b_i_*_,*k*_	1 when vehicle *K* accesses node *i*, otherwise 0
*TCUM_i_*_,*k*_	Wait time of point *i* when vehicle *k* arrives at node *i*, when *b_i_*_,*k*_ = 0, *TCUM_i_*_,*k*_ = 0

**Table 3:** Information of MO-CCVRP-OT.

Constraint Catalog	Constraint Name	Constraints Considered by Our Model
Road network	Symmetry	Symmetric
	Measurement	2-D Euclidean distance
Vehicles	Vehicles type	Homogeneous
	Limitation	Load capacity
Customer (disaster sites)	Priority	Unprioritized
	Visit times	One Visit
	Is good splittable	Unsplittable
Depot	Capacity	Unlimited
	Number	Single
Time Period		One span within a day

**Table 4:** Information on nodes.

No.	x-Coordi- Nates (km)	y-Coordi- Nates (km)	Demands (cwt)	Unloading Time (m)	No.	x-Coordi- Nates (km)	y-Coordi- Nates (km)	Demands (cwt)	Unloading Time (m)
1	30	40	0	0	21	57	58	28	9
2	37	52	7	2	22	62	42	8	3
3	49	49	30	10	23	42	57	8	3
4	52	64	16	5	24	16	57	16	5
5	20	26	9	3	25	8	52	10	3
6	40	30	21	7	26	7	38	28	9
7	21	47	15	5	27	27	68	7	2
8	17	63	19	6	28	30	48	15	5
9	31	62	23	8	29	43	67	14	5
10	52	33	11	4	30	58	48	6	2
11	51	21	5	2	31	58	27	19	6
12	42	41	19	6	32	37	69	11	4
13	31	32	29	10	33	38	46	12	4
14	5	25	23	8	34	46	10	23	8
15	12	42	21	7	35	61	33	26	9
16	36	16	10	3	36	62	63	17	6
17	52	41	15	5	37	63	69	6	2
18	27	23	3	1	38	32	22	9	3
19	17	33	41	14	39	45	35	15	5
20	13	13	9	3	40	59	15	14	5

**Table 5:** Information on solution results of 20 runs of DGWO-F2OPT solving MO-CCVRP-OT.

No.	Number of Non-Dominated Solutions	Running Time (s)
1	1	25.173
2	4	28.4
3	3	22.373
4	6	22.994
5	1	19.64
6	2	20.161
7	2	22.601
8	1	21.28
9	1	20.537
10	1	21.146
11	3	15.821
12	1	21.165
13	1	19.959
14	2	21.205
15	3	19.749
16	2	21.107
17	4	17.026
18	3	17.582
19	5	18.815
20	4	24.985

**Table 6:** Non-dominated solutions in No. 4 solution result.

No.	*fitness*_1_	*fitness*_2_	Denoted by
1	2797	2540	Utopia optimum
2	2723	3380	
3	2999	2420	
4	2895	2500	
5	2759	3000	
6	2737	3180	

**Table 7:** Detailed information for utopia optimum non-dominated solution.

No.	Total Load Capacity (cwt)	Wait Time (Minute)	Operation Time (Minute)	Exceeding (Minute)
1	139	577	163	43
2	115	493	139	19
3	108	593	150	30
4	108	689	155	35
5	68	308	101	0
6	80	137	94	0
Total	618	2797	802	127

**Table 8:** Twenty runs statistical information on solution results of DGWO-2OPT and DGWO.

DGWO-2OPT			DGWO		
No.	Number of Non-Dominated Solutions	Running Time (s)	NO.	Number of Non-Dominated Solutions	Running Time (s)
1	1	19.894	1	2	6.976
2	2	18.901	2	1	6.786
3	1	16.462	3	1	6.924
4	1	17.145	4	1	6.842
5	2	15.91	5	1	6.802
6	1	18.951	6	1	6.886
7	1	17.82	7	1	6.796
8	1	17.233	8	2	6.712
9	1	17.686	9	1	6.618
10	2	17.652	10	1	6.779
11	1	16.988	11	1	6.944
12	2	16.738	12	1	6.791
13	2	18.718	13	1	6.764
14	4	15.536	14	1	6.726
15	2	18.601	15	1	6.764
16	1	16.987	16	1	6.754
17	1	16.871	17	3	6.795
18	2	17.574	18	1	6.66
19	2	16.327	19	1	6.709
20	2	17.34	20	1	6.898

**Table 9:** Average numbers of non-dominated solutions and average runtime of 20 runs.

Algorithm	Average Number of Non-Dominated Solutions	Average Running Time (s)	Average Value of Fitnesses
DGWO	1.2	6.796	6089.5
DGWO-2OPT	1.6	17.467	2845.5
DGWO-F2OPT	2.2	21.086	2668.5

**Table 10:** Comparison of the solutions of CVRP-OT and MO-CCVRP-OT.

Model	CVRP-OT	MO-CCVRP-OT	Deviation (%)
Wait time of vehicles at all disaster-affected points (m)	819.78	811.13	−1.06
Cost incurred by the excess operation time of rescue vehicles (Subsidy unit)	534.4	535.2	0.15
Total travel time (m)	266.72	266.76	0.01
Number of vehicles dispatched	2	2	0
Euclidean distance from the Utopia point	46.16	37.52	−18.72

## Data Availability

The data presented in this study are available on request from the corresponding author.
